# Causality of Blood Metabolites on Proliferative Diabetic Retinopathy: Insights From a Genetic Perspective

**DOI:** 10.1155/2024/6828908

**Published:** 2024-10-30

**Authors:** Zhaoxiang Wang, Bing Lu, Li Zhang, Yuwen Xia, Xiaoping Shao, Shao Zhong

**Affiliations:** ^1^Department of Endocrinology, The First People's Hospital of Kunshan, Kunshan, Jiangsu 215300, China; ^2^Department of Clinical Nutrition, The First People's Hospital of Kunshan, Kunshan, Jiangsu 215300, China

**Keywords:** diabetes complication, Mendelian randomization, metabolites, proliferative diabetic retinopathy, ratios

## Abstract

**Background:** Our goal was to examine the causal link between blood metabolites, their ratios, and the risk of developing proliferative diabetic retinopathy (PDR) from a genetic insight.

**Methods:** Summary-level data about 1400 blood metabolites and their ratios, as well as PDR, were sourced from prior genome-wide association studies (GWAS). A two-sample univariate and multivariate Mendelian randomization (MR) approach was utilized. Additionally, metabolic pathway analysis and sensitivity analysis were also conducted.

**Results:** After adjusting for multiple tests, four blood metabolites significantly correlated with PDR risk. Two ceramides, including glycosyl-N-palmitoyl-sphingosine (d18:1/16:0) (odds ratio [OR] = 1.12, 95% confidence interval (CI): 1.06–1.17, *p* < 0.001, false discovery rate (FDR) = 0.005) and glycosyl-N-behenoyl-sphingadienine (d18:2/22:0) (OR = 1.11, 95% CI: 1.06–1.16, *p* < 0.001, FDR = 0.017), were linked to increased risk. Additionally, 3-methylcytidine (OR = 1.05, 95% CI: 1.03–1.08, *p* < 0.001, FDR = 0.021) also posed a risk, whereas (N(1)+N(8))-acetylspermidine (OR = 0.91, 95% CI: 0.87–0.94, *p* < 0.001, FDR = 0.002) appeared protective. Multivariable MR analysis further confirmed a direct, protective effect of (N(1)+N(8))-acetylspermidine on PDR risk (OR = 0.94, 95% CI: 0.89–1.00, *p* = 0.040). The sensitivity analysis results indicated that evidence for heterogeneity and pleiotropy was absent.

**Conclusion:** These metabolites have the potential to be used as biomarkers and are promising for future research into the mechanisms and drug targets for PDR.

## 1. Introduction

As a chronic and progressive disease, diabetic retinopathy is caused by damage to the retinal microvascular system and stands as a leading cause of vision impairment in working-age adults [1]. PDR marks the advanced stage of diabetic retinopathy, distinguished by the formation of new blood vessels in the retina, leading to irreversible vision loss that defies complete cure [1, 2].

Metabolites, the small molecules generated during metabolic processes, are influenced by genetics, dietary habits, lifestyle, gut microbiome, and various diseases [3]. These molecules can affect the risk of disease and are typically targeted in therapeutic approaches [4]. Thus, identifying the causation role of metabolites in the development of diseases could offer practical intervention targets for therapeutic strategies [5]. Previous metabolomic studies have also identified several potential differential metabolites, such as lipid metabolites ceramides [6]. In diabetic patients, impaired retinal ceramide metabolism leads to increased accumulation, contributing to retinal pathology [7]. However, metabolomic studies on PDR are still in their infancy, with limited published research and reproducibility of findings [8–10].

MR analysis leverages genetic variants as instrumental variables (IVs) to offer a more reliable approach for determining causality compared to traditional observational studies, as it addresses confounding factors more effectively [11]. In this investigation, we undertook a two-sample univariate and multivariate MR analysis to thoroughly examine the causal link between blood metabolites, their ratios, and PDR risk [12].

## 2. Materials and Methods

### 2.1. Data Sources

A pooled dataset on the genetics of 1400 human blood metabolites and their ratios was gathered from the comprehensive GWAS related to metabolomics [3]. The summary statistics about metabolites and their ratios have been archived in the GWAS Catalog (https://www.ebi.ac.uk/gwas/) [13]. The accession numbers for the European dataset are GCST90199621-90201020. The research targeted 8299 unrelated European subjects in the Canadian Longitudinal Study of Aging (CLSA), all of whom underwent genome-wide genotyping and blood metabolite measurement. The CLSA tracks over 50,000 Canadians aged 45–85 for various types of information such as biological and medical data [14]. A sum of 1091 human blood metabolites and 309 their ratios with genetic influences have been identified. These levels of blood metabolite were measured using the ultrahigh performance liquid chromatography–tandem mass spectrometry (UPLC-MS/MS) platform by Metabolon Inc. Given that some metabolites act as substrates and products in enzyme-mediated reactions, pinpointing genetic factors behind the substrate-to-product ratio can reveal biological processes missed when focusing on single metabolites alone. To find these genetic determinants of metabolic flux, 309 metabolite level ratios were calculated for pairs involving the same enzyme or transporter, using data from the Human Metabolome Database (HMDB). The ratio of metabolite pairs was determined by dividing one metabolite's batch-normalized value by the others in the same individual. Summary-level data for PDR GWAS were retrieved from the FinnGen consortium's R9 release [15]. The FinnGen consortium's GWAS data for PDR comprised 9511 cases and 362,581 controls. PDR, identified by retinal neovascularization, represents the advanced stage of diabetic retinopathy (ICD-10 code: H36.03). The FinnGen consortium is a Finnish nationwide GWAS meta-analysis encompassing nine biobanks, with minimal overlap with GWAS of blood metabolites, reducing the risk of bias [15]. Further information about the FinnGen consortium can be found at https://finngen.gitbook.io/documentation/v/r9/.

### 2.2. Selection Criteria for IVs

In our study, IVs were chosen using association thresholds of *p* < 1e − 5, a common practice when limited single nucleotide polymorphisms (SNPs) are available for the exposure in MR analysis [16–18]. To mitigate the influence of SNP associations, linkage disequilibrium analysis (*r*^2^ < 0.1, clumping distance = 500 kb) was adopted with reference to the European 1000 Genomes Project Phase 3 panel [16–18]. At the same time, palindromic SNPs were further excluded to reduce allele variation impacts, and MR Steiger filtering was employed to exclude SNPs with incorrect causal directions. The applicability of IVs for metabolite levels and their ratios was assessed using explained variance (*R*^2^) and *F*-statistic parameters, and IVs with *F*-statistics < 10 were excluded. Finally, the PhenoScanner platform was used to rule out SNPs related to possible confounding variables [19, 20]. The PhenoScanner platform facilitates the exploration of the links between genetic variations and human phenotypes through its web-based interface, which taps into a broad database of numerous studies [19].

### 2.3. MR Analysis

The MR analysis relied on three indispensable assumptions: (1) genetic variation must be reliably related to the exposure studied, (2) genetic variation must be independent of any confounding factors, and (3) genetic variation must impact the outcome studied only through the exposure studied, not through other direct pathways [21]. The analytical methods employed in this study are primarily based on the R software, mainly using packages like “TwoSampleMR”, “MR-PRESSO”, and “MendelianRandomization”. Two-sample MR analyses were employed to evaluate the causal impacts of blood metabolites, their ratios, and PDR risk. Five common MR methods, including inverse variance weighted (IVW), weighted median, simple mode, weighted mode, and MR-Egger regression, were employed [20]. The primary outcome assessment method was the fixed-effects IVW, which integrates the Wald ratios for each SNP. Other methods were utilized to further substantiate the findings. To address and mitigate the potential for false positives that can complicate the outcomes of multiple testing, we employed the false discovery rate (FDR) correction in our MR analyses. For metabolites with an FDR less than 0.05, we conducted a replication MR analysis using the metabolite GWAS summary data provided by Schlosser et al. [22]. Blood metabolites and their ratios presenting a *p* value less than 0.05 without satisfying the FDR significance threshold (FDR < 0.05) were considered to potentially exhibit a causal effect. The robustness of the MR estimates was assessed by sensitivity analyses. The heterogeneity between IVs was measured using Cochrane's *Q* test. When a *p* value less than 0.05 was observed, indicating heterogeneity, random-effect IVW was utilized in place of fixed-effect IVW. The MR-Egger regression intercept served to detect underlying horizontal pleiotropy, with horizontal pleiotropy being suggested by a *p* value < 0.05 [23]. Overall horizontal pleiotropy was assessed using the MR-PRESSO global test, and MR-PRESSO addressed horizontal pleiotropy by removing the outliers. A leave-one-out analysis was carried out to determine the effect of potentially significant IVs by removing single SNPs one by one and calculating the effect using the IVW method. Additionally, we performed multivariable MR analyses, encompassing MVMR-IVW, MVMR-Egger, MVMR-Lasso, and MVMR-Median, to uncover potential vertical pleiotropic pathways of the positive metabolites and ratios [23]. This allowed us to estimate their direct causal effects on PDR risk after mutually adjusting for one another. The parameters were set in alignment with the univariable MR analysis.

### 2.4. Metabolic Pathway Enrichment Analysis

For metabolites with a *p* value < 0.05, we performed a metabolic pathway function analysis using MetaboAnalyst 5.0 (https://www.metaboanalyst.ca/), an online platform designed for efficient analysis of metabolomic data in accordance with the Small Molecule Pathway Database (SMPDB) (https://smpdb.ca/) [24, 25]. SMPDB is a database that visually and interactively presents over 30,000 small molecule pathways [24]. It aids in the exploration and understanding of pathways within metabolomics, transcriptomics, proteomics, and systems biology [24].

## 3. Results

### 3.1. Selection of IVs

Following an extensive quality control procedure, we have discovered a total of 42,348 SNPs associated with 1400 metabolites and their ratios as IVs for investigating the association between metabolites, their ratios, and PDR. Each SNP's *F*-statistic surpassed 10, ensuring the absence of weak IVs. All-encompassing details for each SNP are included in Attachment S1.

### 3.2. Univariable MR Analysis

**Figure 1:**
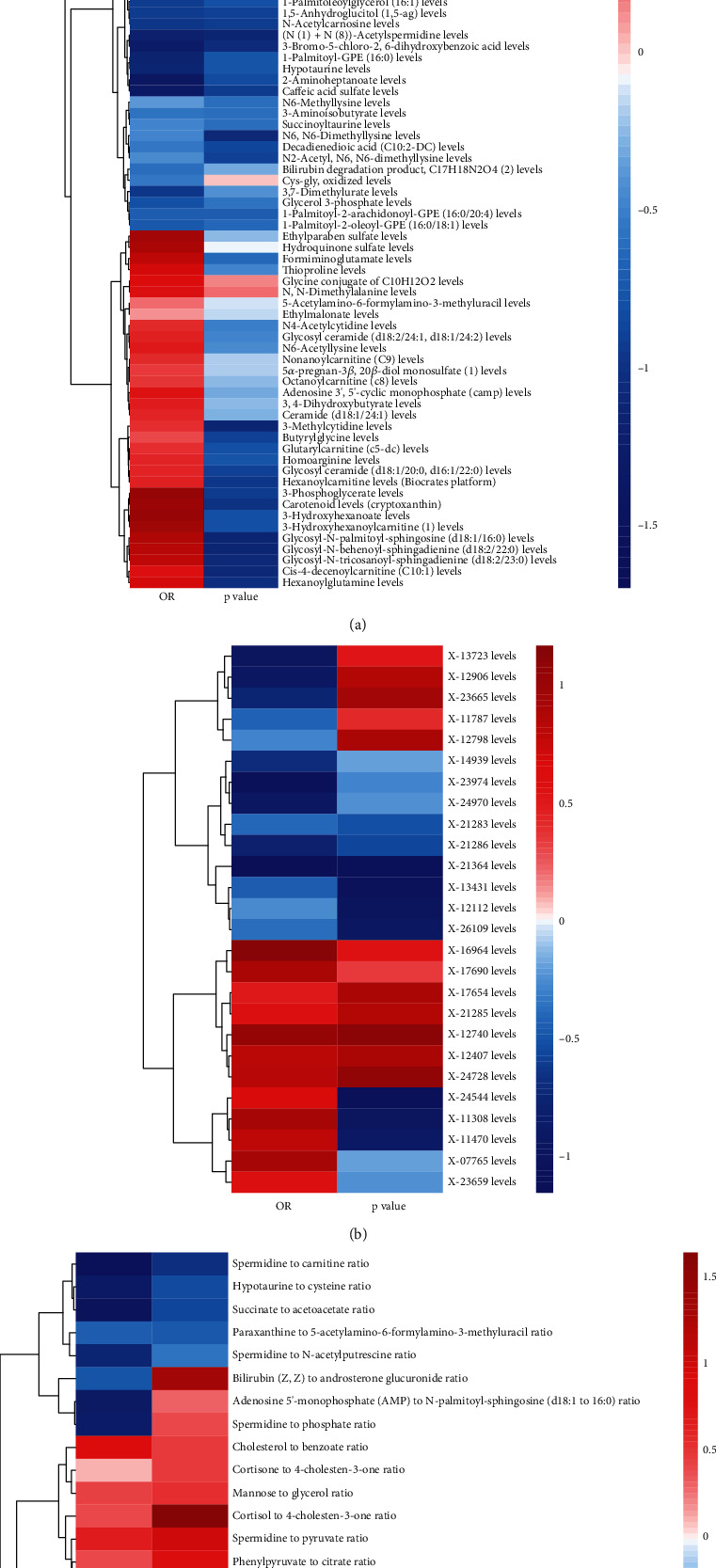
Relationships between metabolites, their ratios, and the risk of PDR: (a) known metabolites; (b) unknown metabolites; (c) metabolite ratios.

**Figure 2:**
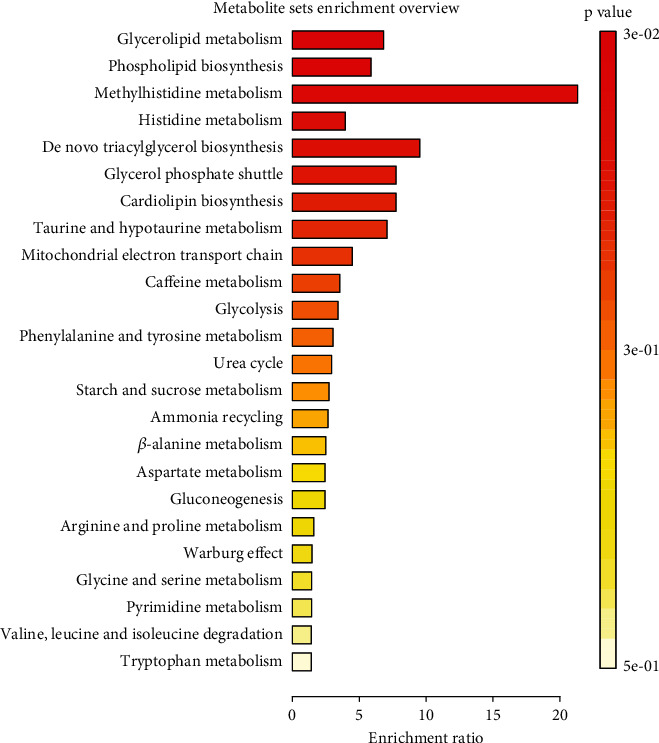
Pathway enrichment analysis results for metabolites.

The results of the comprehensive MR analysis of metabolites, their ratios, and the risk of PDR are detailed in Attachment S2. The results of IVW analysis found a sum of 118 blood metabolites, consisting of 92 known metabolites and 26 unknown metabolites, as well as 25 metabolite ratios that might be related to the risk of PDR (Figures 1(a), 1(b), and 1(c)). Most of these potential metabolite ratios correlate with the differential metabolites identified above, such as N-acetylputrescine-to-(N(1)+N(8))-acetylspermidine ratio, spermidine-to-carnitine ratio, and hypotaurine-to-cysteine ratio. Metabolic pathway function analysis using MetaboAnalyst online platform for the identified known blood metabolites showed that the top-ranked pathways include glycerolipid metabolism, phospholipid biosynthesis, histidine metabolism, and methylhistidine metabolism (Figure 2).

**Figure 3:**
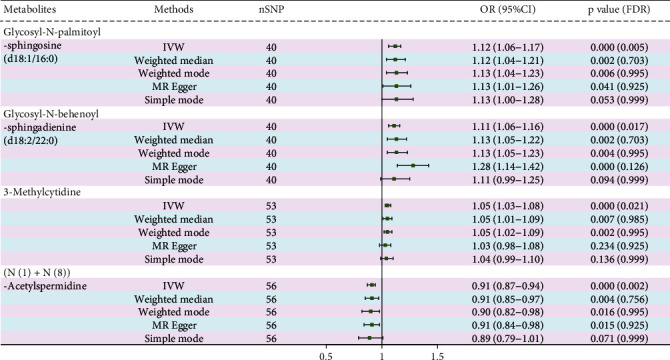
Causal relationships between four metabolites and PDR risk.

**Figure 4:**
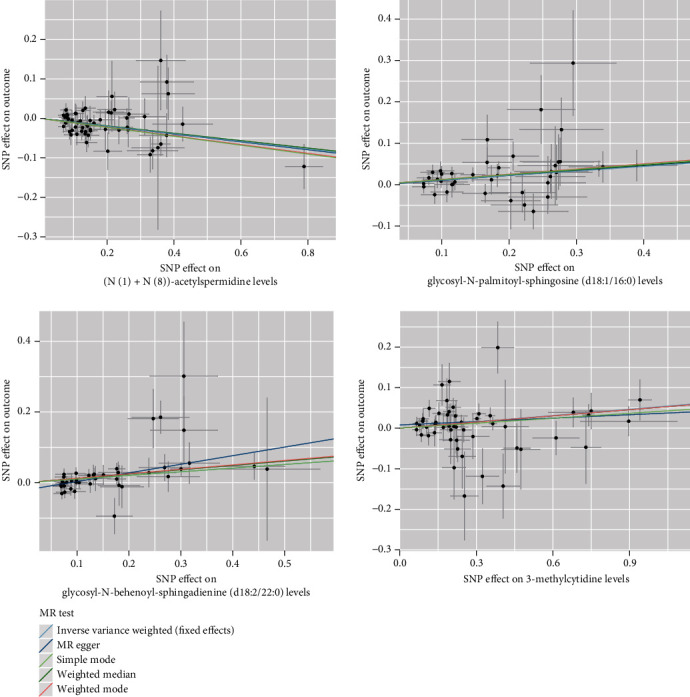
Scatter plots showing the causal impact of four metabolites on PDR risk.

**Figure 5:**
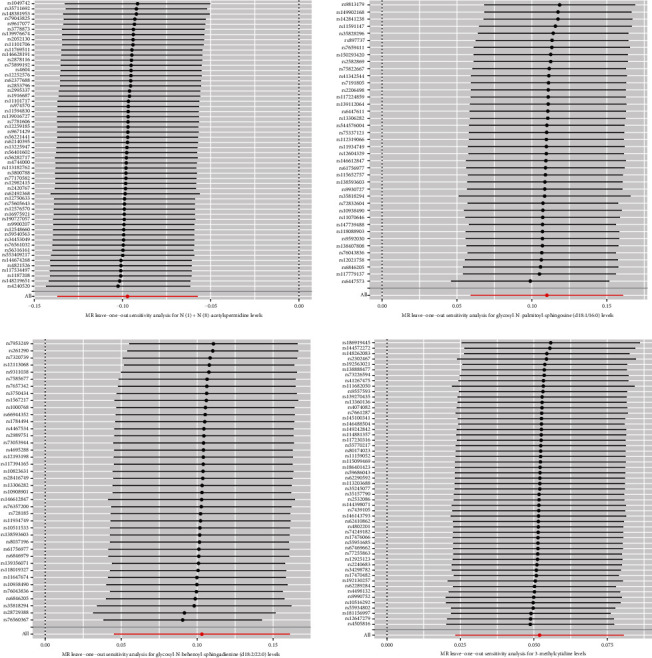
Findings from the leave-one-out method for four metabolites in relation to PDR risk.

After the FDR correction of IVW results, four metabolites were discovered to have a significant causal association with PDR risk (FDR < 0.05) (Figure 3). The results of FDR correction for other MR analysis methods are provided in Attachment S2. The IVW MR estimation indicated a significant positive relationship between glycosyl-N-palmitoyl-sphingosine (d18:1/16:0) (OR = 1.12, 95% CI: 1.06–1.17, *p* < 0.001, FDR = 0.005), glycosyl-N-behenoyl-sphingadienine (d18:2/22:0) (OR = 1.11, 95% CI: 1.06–1.16, *p* < 0.001, FDR = 0.017), 3-methylcytidine (OR = 1.05, 95% CI: 1.03–1.08, *p* < 0.001, FDR = 0.021), and the risk of PDR. Conversely, the levels of (N(1)+N(8))-acetylspermidine (OR = 0.91, 95% CI: 0.87–0.94, *p* < 0.001, FDR = 0.002) indicated a protective effect in reducing PDR risk. The MR analyses using MR-Egger, weighted median, simple mode, and weighted mode also indicated a casual trend.  Figure 4 shows scatter plots of these four metabolites in relation to PDR risk. The results of the sensitivity analysis are also detailed in Attachment S2. The findings from Cochrane's *Q* test suggest evidence for heterogeneity among the SNPs linked to the four blood metabolites in their prediction of PDR risk was absent (*p* > 0.05). The intercept of MR-Egger regression also suggested evidence for pleiotropy was absent (*p* > 0.05). On the other hand, the MR-PRESSO analysis likewise found no significant horizontal pleiotropy (*p* > 0.05). Furthermore, as shown in Figure 5, no influential SNPs affecting the overall effect estimate were identified by the leave-one-out analysis.

### 3.3. Repeated MR Analysis Based on External Dataset

**Figure 6:**
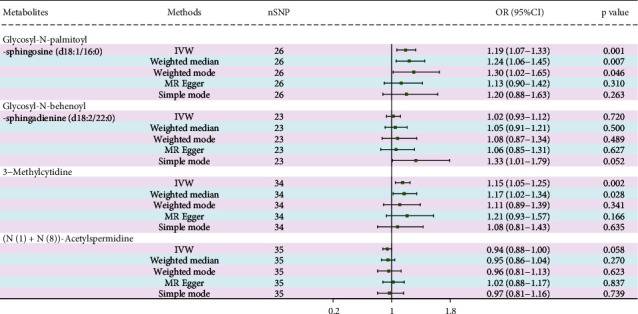
Repeated MR analysis of four metabolites.

Utilizing external metabolite GWAS datasets, we further conducted repeated MR analyses on the four above metabolites (Figure 6). The IVW MR estimation identified a significant positive association between glycosyl-N-palmitoyl-sphingosine (d18:1/16:0) (OR = 1.19, 95% CI: 1.07–1.33, *p* = 0.001) and 3-methylcytidine (OR = 1.15, 95% CI: 1.05–1.25, *p* = 0.002) with PDR risk. Conversely, (N(1)+N(8))-acetylspermidine showed a potential causal trend with PDR risk (OR = 0.94, 95% CI: 0.88–1.00, *p* = 0.058).

### 3.4. Multivariable MR Analysis

**Figure 7:**
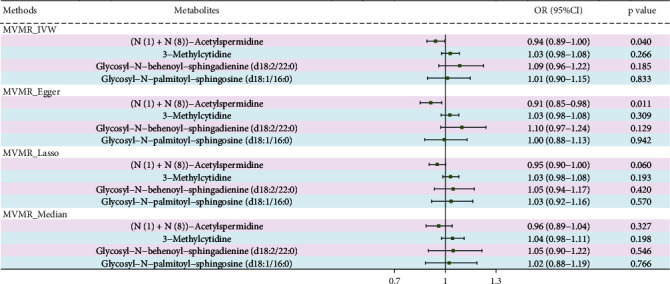
Findings from the multivariable MR analysis.

To evaluate the direct effect of these four metabolites on PDR risk together, a multivariable MR analysis was utilized (Figure 7). The trend of causal direction for these four metabolites aligns with the univariate MR analysis results after mutual adjustment. However, based on the results of MVMR-IVW, we only observed that the levels of (N(1)+N(8))-acetylspermidine have a direct causal link on PDR risk (OR = 0.94, 95% CI: 0.89–1.00, *p* = 0.040). Based on the MVMR_Egger results, we also observed a protective effect of (N(1)+N(8))-acetylspermidine on PDR (OR = 0.91, 95% CI: 0.85–0.98, *p* = 0.011). However, the MVMR_Lasso and MVMR_Median methods only indicated a trend towards a causal effect. The MVMR-Egger intercept results indicate that our multivariable MR estimates are stable and unlikely to be biased by pleiotropy, with a *p* value for the MVMR-Egger intercept of 0.142.

## 4. Discussion

From a genetic perspective, this is the initial MR research examining the causal link between blood metabolites, their ratios, and PDR risk. We innovatively report four novel metabolites associated with the risk of PDR and further highlight the role of ceramide in PDR.

In our research, we confirmed some potential metabolites previously identified in other studies, such as phenylalanine, citrulline, histidine, and piperine, clarifying their potential pathogenic/protective roles [26–29]. On the other hand, we also identified several novel potential metabolites and metabolite ratios. Analysis of blood metabolites identified the top pathways as glycerolipid metabolism, phospholipid biosynthesis, and histidine metabolism. Hyperglycemia can lead to dysregulation of lipid metabolism, with abnormal synthesis and degradation of triglycerides potentially causing an imbalance in the energy supply to retinal cells, thereby exacerbating retinal damage [30]. Changes in the composition of the cell membrane directly affect the function of retinal cells; diabetes may influence phospholipid synthesis, resulting in membrane instability and functional abnormalities [31]. Glycerolipid and phospholipid metabolism have also been hinted at in previous studies of retinal diseases [32, 33]. Moreover, disruptions in the metabolism of histidine and its derivatives (such as histamine) might enhance local inflammatory responses, further aggravating retinal damage [28, 34]. Diabetic patients frequently experience oxidative stress and inflammation, which are closely intertwined with the metabolic pathways, thereby further contributing to retinal cell damage [35]. After multiple testing corrections, we have identified four metabolites with significant causal relationships. It is noteworthy that the two metabolites, glycosyl-N-palmitoyl-sphingosine (d18:1/16:0) and glycosyl-N-behenoyl-sphingadienine (d18:2/22:0), are both glycosylated ceramides, belonging to the sphingolipid family. Dyslipidemia is intricately linked to diabetic retinopathy [6]. Dysregulation of ceramide metabolism in diabetic retinopathy, as a lipid class, is extensively supported by evidence from animal and cell culture studies [36, 37]. During retinopathy, ceramides participate in diverse processes including neuronal survival and death, proliferation and migration of cells, inflammation, and neovascularization [38]. The presence of ceramides further intensifies the hyperglycemia-induced activation of the Rac1-Nox2-ROS signaling pathway and leads to increased mitochondrial damage [7]. Previous studies have shown that short-chain ceramides (*C* ≤ 24) exhibit notable proinflammatory and proapoptotic traits compared to very long-chain ceramides [6]. Moreover, ceramides with C16 and C18 acyl chains provide a better independent prediction for vascular lesions than traditional lipid markers [39–41]. (N(1)+N(8))-acetylspermidine is a polyamine compound made up of N(1)-acetylspermidine and N(8)-acetylspermidine. It is essential in biological processes such as cell metabolism and division and has been discovered to possess antioxidant and anti-inflammatory properties [42–44]. 3-Methylcytidine is a nucleoside composed of a cytidine base and a ribose. It is a common modified nucleoside in RNA and can influence the stability, translation, and degradation of RNA [45]. The role of 3-methylcytidine in diabetic retinopathy remains undefined, but several mechanisms warrant attention. These include its influence on RNA modifications, potential regulation of oxidative stress, and involvement in disease progression by modulating RNA-associated inflammatory pathways [46–48].

The interpretation of the results might be affected by several limitations within this study. Firstly, the SNPs employed failed to meet the GWAS significance criterion of 5e − 8. Although a relatively lenient threshold of 1e − 5 was used, it is also regarded as a standard for blood metabolites [49, 50]. Secondly, this study's participant pool was mostly of European origin, which lessened the population diversity. Additionally, although we validated the four metabolites with external metabolite datasets, no suitable external PDR datasets were available for further repeated MR analysis. Thirdly, this study's findings encompassed numerous associations (potential causal metabolites) that were not significant by FDR standards. Pathway analysis of these nonsignificant results might also yield false positives. Fourthly, although FDR correction confirmed causal metabolites in our IVW analysis, the results from other MR methods were inconsistent after FDR adjustment, showing only a causal trend. Lastly, while the MR approach is valuable for inferring causality, further molecular biology experiments and mechanistic studies are also needed.

## 5. Conclusion

From a genetic standpoint, we pinpointed several metabolites and ratios closely linked to PDR that could act as biomarkers. They might help to provide a promising direction for future research focused on understanding the mechanisms and discovering new drug targets for PDR.

## Data Availability

The pooled dataset in our research originated from the publicly available databases, including https://www.ebi.ac.uk/gwas/, https://finngen.gitbook.io/documentation/v/r9/, and https://smpdb.ca/.

## Supporting Information

Supporting Information Additional supporting information can be found online in the Supporting Information section. Attachment S1. Detailed information about IVs. Attachment S2. Detailed MR analysis results of blood metabolites and their ratios.
