# Supplementary material for: Causality of Blood Metabolites on Proliferative Diabetic Retinopathy: Insights From a Genetic Perspective
Source: J Diabetes Res. 2024 Oct 30;2024:6828908. doi: 10.1155/2024/6828908 (PMC11540900; doi:10.1155/2024/6828908)
Supplement: Supporting Information — Additional supporting information can be found online in the Supporting Information section. Attachment S1. Detailed information about IVs. Attachment S2. Detailed MR analysis results of blood metabolites and their ratios. [file 6828908.f1.docx]

**Supplemental materials**

The supplementary materials (Attachments 1-2) for this article could be accessed in the Figshare database (<https://doi.org/10.6084/m9.figshare.24784446.v2>).
